# Typho-Malarial Fever

**Published:** 1891-04

**Authors:** E. H. M. Parham

**Affiliations:** Fordyce, Ark.


### TYPHO-MALARIAL FEVER.

BY E. H. M. PARHAM, M. D., FORDYCE, ARK.

I am aware that the above is claimed to be a misnomer, for a form of continued fever, prevalent almost everywhere, and appearing at all seasons of the year, but I know that it is one that expends its force on the bowels, and such remedies as are calculated to check or favorably modify its course, should, if possible, be timely used. I am not prepared to determine whether the typhoid bacilli can be found in this disease or not, as I have neither the means nor the time to make a thorough investigation. Let that be as it may, I have often found iodine and carbolic acid to greatly disturb the digestive functions, and their use had to be abandoned, and can not call to mind a single instance that good results could be referred to them. Therefore we must conclude that the typhoid bacilli do not exist in such cases, or iodine and carbolic acid (known to be antagonistic to their propagation, or even existence) would be attended with good results

Symptoms The subject of this disease usually complains at least a week, of indisposition to attend to business of any kind; restlessness, disturbed sleep, with unpleasant dreams, uneasy sensations in his stomach and bowels, total loss of appetite; and these symptoms continue to grow worse until he is forced to apply for medical advice.

On physical examination we now find slight tenderness over the regions of the liver, stomach and spleen, sometimes also in the illiac regions, the tongue coated with white fur, and pale with indentions on the edges; the urine very red, and the dejections from the bowels light colored and thin, and the discharges frequent; at this time the temperature ranges from 101 to 103; pulse from 90 to 100. If these symptoms continue over two weeks we have reasons to fear profuse hemorrhage from the bowels. We rarely ever find any skin eruption, characteristic of typhoid fever.

This is the first, or abortive stage; however, should the remedies used fail to make a favorable impression, about the 15th. day, we may expect to find the toungue dry, pointed and red; some delirium at night, pulse from 100 to 110; temperature 103 in the morning and 104 in the evening. About this time there is always danger of hemorrhage from the bowels, which marks the critical period of this disease. At this time the patient is always very weak, and often needs support, both from diet and stimulation. Sometimes nasal hemorrhage supersedes that of the bowels, and by relieving the congested capillaries, seems to be attended with favorable results, provided always, that we can control the hemorrhage in due time. It is well to remark here, that the abdomen is found tympanitic in this stage, also increased tenderness, and almost always, a gurgling noise can be distinctly heard in the illiac regions, and sometimes may be heard in other portions of the bowels.

Treatment. If called to see the patient in the first stage of the fever, I commence the treatment with the following:

℞ Calomel, gr. v.
Dover's powder, gr. x.

Mix and make 4 powders. Sig. One every three hours.

If this fails to move the bowels, I order a small dose of castor oil, with 10 drops of oil of turpentine. I use the above prescription to correct morbid secretions, and thereby place the patient in a better condition to receive further treatment; should this be immediately followed by a decided remission, I order three grains of quinine every two hours until twenty grains, are taken, which seems to me, to make an impression on this disease, directly calculated to make it run a milder course even though it fails to abort it.

In the second stage, I abandon the mercurial and quinine treatment, and commence the following:

R Tinct. digitalis.
Tinct. gelsemium, aa f℥ i.
Sweet spts. of nitre, f℥ 1 1-2.

Mix. Sig. Teaspoonful every six hours.

I alternate the above with an emulsion of the following ingredients.

R Spts. of turpentine, f ℥ j.

Tinct. of opium, f ℥ ss.

Comp. spts. of lavender, f ℥ j.

Gum arabic, f ℥ iij.

Water, f ℥ jss.

Mix. Sig. Teaspoonful as above directed.

I reduce the high temperature by the application of cold water to the bowels, "pro re nata," as a safe and efficient remedy, and condemn in decided terms, the antipyretics, such as antipyrine, antifebrine, &c., as calculated to deprive the patient of strength, without any curative tendency.

Should hemorrhage from the bowels occur, I prescribe the following enema:

℞ Powdered alum, gr. xx
Tanic acid, gr. x.
Tinct. opium, gr. xxx.
Warm water, f ℥ ij.

Mix and use for one injection.

I also use ergotole in ten drop doses, either per orem or hypodermically. The injections and ergot (at short intervals, according to the severity of the case) should be continued until the hemorrhage ceases.

If the discharges are watery, and exhaustive, at any stage of the disease, I give the following powders after each action until the bowels are checked:

℞ Sub nitrate of bismuth, gr. xx.
Tannic acid, gr. x.
Powdered opium, gr. iij.

Mix and divide into 8 powders, and use as above directed. If the patient at any time is found sleeping quietly, I do not allow him or her to be awakened, even for the administration of medicine.

If the vital powers at any time seems to be failing, I do not hesitate to stimulate according to the necessity of the case. The diet should be light, but nourishing, such as milk, coffee, &c.

I have written a synopsis of the treatment of the above named disease, which in my hands has been very satisfactory' having lost not more than 3 per cent. of all the cases thus treated.

